# Is it time for Canada to revisit its approach to prostate cancer screening?

**DOI:** 10.1016/j.lana.2025.101180

**Published:** 2025-07-24

**Authors:** David-Dan Nguyen, Aisha Lofters, Christopher J.D. Wallis, Alexandre R. Zlotta, Neil E. Fleshner, Quoc-Dien Trinh, Antonio Finelli, Laura C. Rosella, Allan S. Detsky, Monique J. Roobol, Girish S. Kulkarni

**Affiliations:** a Division of Urology, Department of Surgery, University of Toronto, ON, Canada; b Institute of Health Policy, Management and Evaluation, Dalla Lana School of Public Health, University of Toronto, ON, Canada; c Department of Family and Community Medicine, Women’s College Hospital, University of Toronto, Toronto, ON, Canada; d Division of Urology, Department of Surgery, Mount Sinai Hospital, Toronto, ON, Canada; e Divisions of Urology and Surgical Oncology, Department of Surgery, Princess Margaret Cancer Center, Toronto, ON, Canada; f Department of Urology, University of Pittsburgh School of Medicine, Pittsburgh, PA, USA; g Department of Epidemiology, Dalla Lana School of Public Health, University of Toronto, ON, Canada; h Department of Medicine, Mount Sinai Hospital & University Health Network, Toronto, ON, Canada; i Department of Medicine, Temerty Faculty of Medicine, University of Toronto, Toronto, ON, Canada; j Department of Urology, Erasmus MC Cancer Institute, Erasmus University Medical Center, Rotterdam, the Netherlands

**Keywords:** Prostate cancer, Screening, Organized, Policy

## Abstract

Prostate cancer is the third leading cause of cancer death among Canadian men. Despite advances in the last decade mitigating overdiagnosis and overtreatment, Canadian guidelines have recommended against routine prostate-specific antigen (PSA) screening since 2014. This has resulted in opportunistic screening, marked by inequitable access, low-value testing, and missed opportunities for early detection. We review global policy developments, emerging trial data, and implementation strategies, which suggest that organised, risk-stratified screening may improve outcomes and equity. However, overdiagnosis and associated harms remain a concern within organised programs. To address this uncertainty and generate timely, policy-relevant evidence, we propose implementing population-wide, adaptive platform trials embedded in the healthcare system. This design would enable real-time integration of new technologies, standardised protocols, and equitable access—hallmarks of a learning healthcare system. Such a model could help Canada modernise prostate cancer screening while carefully weighing benefits, harms, and equity in a rapidly evolving landscape.

## Introduction

The use of the prostate-specific antigen (PSA) test as a screening test for prostate cancer was once heralded as a breakthrough in prostate cancer early detection. This simple blood test appeared to dramatically reduce cases of metastatic disease at presentation.^1^ Yet, as data accumulated, PSA screening revealed itself as a double-edged sword. It often caught tumours that posed little risk, leading to a cascade of avoidable interventions with serious, life-altering side effects, including biopsy-associated sepsis, incontinence, and impotence.^2^ The harm of widespread screening prompted health authorities—including Canada in 2014—to reverse course and advise against routine PSA screening.^3^

A decade later, emerging evidence and shifting global perspectives—including recent calls from the *Lancet Commission on Prostate Cancer* to rethink and modernise screening^4^—have motivated us to re-examine Canada’s current approach. In this article, we review new evidence and global policy developments since 2014, examine the unintended consequences of this ongoing stance, and propose an organised and adaptive platform trial approach to prostate cancer screening in Canada.

## Why is prostate cancer screening not recommended?

The 2014 Canadian Task Force on Preventive Health Care and other guidelines groups from that era highlighted two primary concerns regarding prostate cancer screening: 1) overdiagnosis and overtreatment; and 2) conflicting evidence of a mortality benefit. As a result, the Canadian Task Force on Preventive Health Care issued a weak recommendation against routine PSA screening for men aged 55 to 69, and a strong recommendation against screening for men younger than 55 or older than 70. Today, evolving practices and emerging data compel us to reexamine these issues, especially among individuals between 55 and 69.

Concerns about overdiagnosis and overtreatment remain pivotal. Overdiagnosis refers to the detection of cancer that would not have caused symptoms or death during a person’s lifetime if it had not been found through screening or incidental testing. Overdiagnosis and its harms are exacerbated when low-value screening occurs in patients less likely to benefit, such as those over 70 or with less than 10 years of life expectancy.^5^^,^^6^ Overtreatment involves unnecessary interventions stemming from overdiagnosis, leading to avoidable complications such as sepsis following prostate biopsy and reduced quality of life secondary to incontinence and impotence following radiation and surgery.^7^ In 2012, it was estimated that the harms of annual PSA screening would be the overdiagnosis and overtreatment of 45 cases and the loss of 1134 life-years free of prostate cancer (lead time years) over the lifetime of 1000 men.^8^

The uncoupling of screening with invasive investigation and treatment has mitigated overdiagnosis and overtreatment and their related complications. Canada has been at the forefront of advances in the field, including integrating pre-biopsy magnetic resonance imaging (MRI) and microultrasonography to enhance detection and reduce unnecessary biopsies.^9^, ^10^, ^11^, ^12^ New prostate biopsy techniques and peri-procedure antimicrobial prophylaxis have substantially reduced the risk of sepsis—a major concern associated with traditional protocols.^13^^,^^14^ Real-world data from the UK support these evolving practices, showing a 79% reduction in harm related to the diagnostic process.^15^ Today, should a person be diagnosed with low-risk prostate cancer, they would be managed by active surveillance rather than radiation or surgery.^16^ To be sure, active surveillance doesn’t eliminate the issue of overdiagnosis—but it does mitigate its consequences. Patients on active surveillance avoid treatment-related side effects, maintain a good quality of life, and, contrary to common assumptions, report favourable levels of anxiety.^17^^,^^18^ Management strategies for localised prostate cancer continue to evolve. There is growing interest in expanding the indications for active surveillance beyond low-risk disease to select patients with favourable intermediate-risk prostate cancer.^19^ In parallel, emerging focal therapy approaches are being investigated as a less invasive alternative to definitive treatment. While still considered investigational, focal therapy may eventually offer an option for patients seeking to balance oncologic control with preservation of quality of life, especially as evidence from ongoing trials matures.^20^^,^^21^ There are also ongoing discussions led by urologists, radiation oncologists, and pathologists about reclassifying certain low-risk prostate cancers as non-cancerous, a significant departure from past practices, where such cases were routinely overtreated.^22^

At the time of the 2014 recommendation, there was scepticism regarding the prostate cancer mortality benefit of PSA screening. The Prostate, Lung, Colorectal, and Ovary (PLCO) cancer screening trial in the United States showed no significant reduction in prostate-cancer-specific mortality associated with PSA screening.^23^ This stood in contrast to the European Randomized study of Screening for Prostate Cancer (ERSPC), which found a significant reduction in prostate cancer deaths with screening but with a high risk of overdiagnosis.^24^

However, the PLCO trial had important limitations which revealed themselves over time and with new data. While contamination in the control arm was acknowledged in 2014, its extent was not fully understood until it became clear that up to 90% of participants in the control group had undergone at least one PSA test during the study—remarkably, the control arm reported higher rates of PSA screening than the intervention arm.^25^ When differences in design, implementation, and setting are accounted for, modelling studies suggest that the ERSPC and PLCO provide evidence that screening provides a relative reduction of 30% in the rate of death from prostate cancer.^26^ Moreover, the ERSPC’s extended follow-up data published in 2014 and 2019 revealed greater long-term prostate-cancer-specific mortality benefits.^27^^,^^28^ The difference in absolute PCa mortality increased from 0.14% at 13 years to 0.18% at 16 years, and is projected to increase over time, considering the long natural history of prostate cancer and the fact that most prostate cancer deaths occur many years after diagnosis. This represents a number-needed-to-invite-to-screen to prevent one prostate cancer death at 16 years of 570, lower than what was reported in 2014 when the number-needed-to-invite-to-screen was estimated to be 742.^27^ By comparison, the Canadian Task Force on Preventive Health Care reports a number-needed-to-screen of 1333 to prevent one breast cancer death among women aged 50–59 years using mammography and a number-needed-to-screen of 377 to prevent one colon cancer death using guaiac faecal occult blood testing.^29^^,^^30^ These recommendations have resulted in organised breast and colorectal cancer screening programs in Canada. Nonetheless, it is crucial to emphasize that overdiagnosis remains significantly more common with traditional PSA screening than with breast or colorectal cancer screening.

Guideline authors also questioned whether prostate cancer screening reduces overall mortality as opposed to prostate-cancer-specific mortality.^3^ The two largest trials to date—the ERSPC with 182,000 participants and the PLCO with 76,693— were unable to provide a definitive answer, largely due to the overwhelming influence of deaths unrelated to prostate cancer.^31^ Testing all-cause mortality in trials for any individual cancer is not feasible because cancers only account for a small proportion of all deaths. Detecting a statistically significant effect on all-cause mortality would require trials exceeding one million participants for cancers such as breast, colorectal, and prostate.^32^ In a meta-analysis of 18 long-term randomised clinical trials involving 2.1 million individuals, there was no compelling evidence that commonly used cancer screening tests—such as PSA testing for prostate cancer, mammography for breast cancer, and colonoscopy or faecal occult blood testing for colorectal cancer—prolong overall lifespan.^33^

Given these updated data and practices, it would be reasonable to revisit the current Canadian recommendations and explore novel approaches to organised screening. In 2018, the U.S. Preventive Services Task Force revised its earlier statement against prostate cancer screening. It now supports shared decision-making for men aged 55 to 69—a position echoed by other North American organizations like the Canadian Urological Association, the American Urological Association, the National Comprehensive Cancer Network, and the American Cancer Society (Table 1).^37^, ^34^, ^36^, ^38^, ^35^ The Canadian Task Force on Preventive Health Care has not changed its statement against prostate cancer screening since 2014. The continued reliance on opportunistic screening has resulted in inconsistent access, persistent inequities, and limited clinical benefit—failing to both take advantage of and evaluate advances that could make prostate cancer screening safer, more effective, and more equitable. We discuss the consequences of the status quo in the next two sections.

**Table 1:** Prostate cancer screening recommendations from major North American public health and urologic organizations.

Organization	Year	Recommendation	Strength of Recommendation
US Preventive Services Task Force^34^	2018	**40–54** Insufficient evidence **55–69** The decision to be screened for prostate cancer should be an individual one **≥70** Do not screen for PCa	Grade C (Offer or provide this service for selected patients depending on individual circumstances) Grade D (Discourage the use of this service)
Canadian Task Force on Preventive Health Care^3^	2014	**40–54** Do not screen for PCa **55–69** Do not screen for PCa **≥70** Do not screen for PCa	Strong (Most individuals will be best served by the recommended course of action) Weak (most men would want the recommended course of action but many would not) Strong
American Urological Association^35^	2023	**40–45** Clinicians should engage in SDM and offer PCa screening to men at increased risk (Black ancestry, germline variations, strong family history of PCa) **45–50** Clinicians may begin prostate cancer screening and offer a baseline PSA test to people between ages 45–50 years **50–69** Clinicians should engage in SDM and offer PCa screening **≥70** Clinicians may decide to discontinue screening based on patient preference, age, PSA, PCa risk, life expectancy, or general health, after SDM	Strong Conditional Strong Conditional
Canadian Urological Association^36^	2022	**45–49** Clinicians should engage in SDM and offer PCa screening to individuals at increased risk **50-Discontinuation** Clinicians should engage in SDM and offer PCa screening **Discontinuation** a. For men aged 60 with a PSA <1 ng/ml b. For all other men, discontinue PSA screening at age 70 c. For men with a life expectancy less than 10 years, discontinue PSA screening	Grade C Grade C Grade C

PCa = Prostate cancer; PSA = Prostate-specific antigen; SDM = Shared decision-making.

## How is the status quo exacerbating disparities and low-value screening?

In the current setting of countervailing guidelines and emphasis on shared decision-making, there is significant clinical variability in screening, resulting in health inequities and inefficiencies.^39^ Opportunistic screening prevails, relying on asymptomatic patients engaging in shared decision-making with their primary care provider on a case-by-case basis. In practice, patients face several hurdles, including having access to a regular primary care provider, navigating and often initiating the decision-making process, and paying out-of-pocket for a PSA test in certain Canadian provinces.

Opportunistic screening thus favours individuals with better access to healthcare, higher levels of health literacy and more disposable income,^40^^,^^41^ inevitably exacerbating disparities along social determinants of health. For instance, Black and Métis patients in the city of Calgary have lower PSA testing rates compared to the general population, while a higher household income and university education are associated with increased testing frequency.^42^ In a provincial analysis, Indigenous individuals in the province of Alberta were less likely to undergo PSA testing and, when diagnosed, had more aggressive tumours at presentation and were more likely to develop metastatic disease than their non-Indigenous counterparts.^43^ In their review of high-income countries, Vickers et al. noted similar inequities along the social determinants of health in prostate cancer screening in the US, UK, and Switzerland, indicating that disparities exist across a broad spectrum of high-income healthcare systems.^40^

Paradoxically, the global review by Vickers et al. also noted that current guidelines have increased low-value screening (screening in individuals unlikely to benefit; e.g., PSA tests after age 70 or in those with limited life expectancy).^40^ Despite consensus against PSA screening for men over 70 or with less than 10 years of life expectancy, studies show it remains common in high-income countries such as the UK, Italy, France, and Germany. In the UK, men aged 80–89 are twice as likely to receive a PSA test as those in their 50s,^44^ and in Italy, half of men over 70 are screened.^45^ While contemporary Canadian data on low-value prostate cancer screening is lacking, in 2015, it was estimated that the province of Ontario spent close to $22 million annually to screen men younger than 50 and older than 74 years of age.^46^

## How have current screening policies affected prostate cancer outcomes?

Prostate cancer remains the most common cancer and the third-leading cause of cancer death among Canadian men.^47^ Data on contemporary trends in prostate cancer stage at diagnosis in Canada are lacking. International data suggest that recommendations against prostate cancer screening may be linked to a higher incidence of metastatic disease at diagnosis.^48^, ^49^, ^50^

Despite the possible increase in metastatic cases at diagnosis, mortality trends vary. For example, data from Ontario indicate that survival has declined for prostate cancer, yet has improved for screened cancers like colon cancer.^51^ On the other hand, national projections suggest stagnant prostate-cancer-specific mortality rates.^47^ This apparent paradox—possible rising metastatic disease yet stable survival—must be understood in the context of significant advances in systemic treatments for metastatic prostate cancer, which have substantially extended survival.^52^ It has been hypothesized that these therapeutic gains are being offset by the negative effects of delayed diagnoses and increasing incurable, metastatic disease at presentation.^48^ While reducing mortality is the ultimate goal of screening, preventing metastatic disease—and the need for costly, non-curative treatments with significant side effects—should not be dismissed, as metastases are a well-recognised surrogate for prostate cancer mortality.^53^ Further, these therapies, though life-prolonging, come at a steep cost both financially and as regards to quality of life.^52^

## How can organised prostate cancer screening address these issues?

Globally, there is growing interest in organised prostate cancer screening. Organised screening programs are designed and managed by health services to promote equitable access to screening and ensure standardized care for all individuals. These programs aim to guarantee timely and consistent follow-up for those with abnormal test results while minimising unnecessary screening or follow-up for those unlikely to benefit.^54^ Global interest in organised screening is driven by rising rates of advanced and metastatic disease at diagnosis, increased prostate cancer mortality, and persistent disparities in outcomes. In addition to reducing advanced-stage diagnoses and prostate cancer mortality,^26^ recent studies suggest that *organised* prostate cancer screening can additionally reduce overdiagnosis and mitigate disparities along the social determinants of health.

For instance, the Prostate Cancer Awareness and Initiative for Screening in the European Union (PRAISE-U), launched in response to the European Union’s recommendation to assess the feasibility of organised prostate cancer screening,^55^ has proposed a risk-stratified organised screening model.^56^ Individuals within a specific age range would be invited to undergo PSA testing, with clearly defined management pathways based on the results. Patients with low PSA levels might forgo additional follow-up, while those with elevated levels—determined by age-specific thresholds—would undergo MRI to refine risk stratification and guide further management. Similarly, the Lancet Commission on Prostate Cancer has emphasized implementing PSA-based, risk-stratified early detection programs augmented by MRI, particularly targeting high-risk populations.^4^ By tailoring the approach, precision screening strategies can reserve invasive treatments for individuals most likely to benefit, thus improving outcomes while addressing the harms of overdiagnosis and overtreatment.

Organised prostate cancer screening can also mitigate inequities. In the Swedish arm of the ERSPC trial, a secondary analysis with 18 years of follow-up showed that organised prostate cancer screening eliminated disparities in mortality among individuals with lower education levels.^57^ This was likely due to fewer barriers to early diagnosis for those of lower socioeconomic status. Thus, even in a public healthcare system like Sweden’s, the equity benefits of organised screening were evident. It is therefore reasonable to assume that such benefits would be achieved in the Canadian healthcare system, where similar barriers exist in addition to significant challenges in access to primary care.^58^

Organised prostate cancer screening could substantially reduce low-value testing by prioritising patients most likely to benefit. Implementing firm eligibility criteria rather than relying solely on shared decision-making ensures that screening targets patients who stand to gain the most. Countries that have implemented a national early detection program for prostate cancer report an almost 80% reduction in low-value PSA testing.^59^

Beyond organised screening, another alternative to the current status quo of opportunistic screening is to eliminate screening altogether.^40^ While this approach may be equitable in access—since no one can obtain screening—it forfeits the potential benefits of early detection, particularly for those at the highest risk who might stand to gain the most from early detection.^60^, ^61^, ^62^

## How should provinces design their organised screening program?

While existing evidence suggests that organised prostate cancer screening has potential advantages,^26^^,^^57^ there is sufficient equipoise to justify the need for ongoing evaluation. Overdiagnosis and overtreatment remain significant concerns of organised prostate cancer screening, as trials like the ERSPC did not incorporate contemporary strategies such as MRI or biomarker-based risk stratification, which show promise in reducing these harms.^24^ This equipoise is reflected in the heterogeneous recommendations across organizations (Table 1) and ongoing trials exploring alternative screening strategies.

Given this equipoise and the active investigation into strategies that balance early detection with minimizing harm, we propose that provincial organised prostate cancer screening programs be designed as population-wide clinical trials integrated into the healthcare system. Such a framework would offer the essential infrastructure for systematic and ongoing assessment of adherence to standardized protocols and screening outcomes. It would also provide better access to easily scalable novel prostate cancer early detection pathways. Linked with population-based registries, such trials would enable precise tracking of patient outcomes, ensuring that screening and subsequent interventions are equitably applied to target populations. A potential control arm, whether no screening or opportunistic screening, would allow for evaluation of the true benefit of organised screening.

However, the design of the treatment arms for this type of screening program and its trials must account for the near quarter-century required for prostate cancer screening trials to reach their primary endpoints and translate into policy. In that time, new protocols, technologies, and approaches may emerge yet remain unevaluated and unimplemented in a traditional trial. For example, the Cluster Randomized Trial of PSA Testing for Prostate Cancer (CAP) published its 15-year data in April 2024 and demonstrated a small reduction in prostate cancer deaths at a median follow-up of 15 years.^63^ Regardless, these data are considered obsolete because a low-intensity screening intervention involving a single invitation for a PSA screening test would not be typical of modern organised screening programs and newer diagnostic methods were not incorporated in the trial.^63^ The ProScreen trial, which integrates the novel 4-kallikrein panel blood test and MRI in the diagnostic pathway, recently reported promising early findings with reduced overdiagnosis of low-grade cancers.^64^ However, the trial's primary endpoint will not be reached until 2035, raising concerns that significant advancements in prostate cancer care may go unassessed in the interim.

An adaptive platform trial design may address the limitations of conventional trials.^65^ This trial design focuses on the disease rather than a specific intervention. It allows for multiple treatment groups, the addition of new experimental treatments, and response-adaptive allocation. Although the challenge of a long lag time between screening and definitive outcomes, such as cancer mortality, is shared with conventional trials, adaptive trials mitigate the risk of outdated conclusions by continuously integrating their own findings and those of other concurrent studies. This ensures that the protocols remain relevant and effective while being directly generalizable to the diverse Canadian population. For instance, the program could begin by randomly inviting patients to risk-stratified PSA screening with subsequent MRI or to usual care (no invitation). If interim results favour one arm, more patients could be allocated to it. If novel biomarkers and polygenic risk scores emerge, new arms combining MRI with these advances could be added. A large adaptive trial would align seamlessly with the core principles of a learning healthcare system, which prioritizes the real-time use of evolving data to refine healthcare delivery.^66^ Canada’s provinces, as quasi-single-payer systems, are in a unique position to lead such an endeavour, unlike other fragmented healthcare systems. Centralized administration of screening invitations, testing, and follow-up via provincial health authorities would enable coordinated implementation and oversight. This centralization is critical for mitigating contamination, a common threat to screening trials in settings with high levels of opportunistic testing. By controlling access to publicly funded screening and systematically documenting participation, provinces could reduce crossover between trial arms and preserve the internal validity of findings. In parallel with these operational advantages, embedding equity-focused mechanisms from the outset is also essential. This includes: (1) setting inclusion targets for historically underrepresented populations such as Indigenous, Black, and immigrant communities; (2) incorporating tailored outreach and recruitment strategies informed by community engagement and stakeholders; and (3) conducting real-time equity audits to monitor participation,^67^ outcomes, and access by race, ethnicity, socioeconomic status, and geography. By integrating these elements into trial governance and design, the platform trial could serve not only as a model for adaptive evidence generation but also as a model for equitable implementation—addressing persistent representativeness gaps seen in prior trials. Canada’s population is among the most demographically diverse in the world. This provides an opportunity to design a platform trial that is not only representative, but also capable of addressing longstanding gaps in the inclusion of racialized, immigrant, and socioeconomically disadvantaged populations, amongst other groups.

A major limitation of this proposal is cost. However, the startup costs of an adaptive trial may be lower than expected, given that Ontario already has established infrastructure for population-based cancer screening through programs for breast, cervical, and colorectal cancer. These existing structures—such as centralized invitation systems, data collection platforms, and performance monitoring—can be leveraged to implement prostate cancer screening trials. Moreover, because cancer incidence and outcomes are routinely tracked through provincial registries, many of the key endpoints are already being measured, which further reduces the marginal cost of trial implementation. A comparable large adaptive platform trial for the early detection of prostate cancer, the TRANSFORM study, is currently underway in the UK, receiving substantial backing from the National Health Service and other funders.^68^ While the UK TRANSFORM trial provides valuable insights, its findings cannot fully account for Canada’s unique demographic and geographic challenges. Conducting a Canadian trial would generate essential local evidence, capacity and engagement, ensuring timely and scalable access and adherence to novel screening strategies within the realities of Canada’s healthcare system. This is particularly relevant given that novel risk-stratification strategies, such as upfront MRI or the use of polygenic risk scores to guide screening,^69^^,^^70^ may not be feasible or generalizable in the Canadian setting due to differences in healthcare infrastructure and genetic ancestry.

We summarize the differences between our proposal and current opportunistic screening in Table 2 and provide an example of a possible design of an adaptive platform trial of prostate cancer screening in Canada in Table 3.

**Table 2:** Comparison of opportunistic screening to an adaptive platform trial approach to prostate cancer screening in Canada.

	Opportunistic screening	Organized screening program incorporating an adaptive platform trial
Equity of access	Access can be uneven, with disparities based on geographic, socioeconomic, and provider differences.	Centralized and standardized criteria promote equitable access across patient populations and geographic locations.
Value of screening	Value varies and depends on providers’ and patients’ individualized decision-making.	Screening is targeted to populations most likely to benefit based on centralized protocols and tools.
Consistency in screening protocols	Protocols vary widely based on provider preferences, locally available resources, and local guidelines, leading to inconsistencies.	Screening follows a uniform protocol, ensuring consistency across all sites participating in the trial.
Monitoring and quality control	No centralized system for quality control; outcomes and complications may vary between sites.	Centralized monitoring and data collection ensure quality control, with outcomes tracked and analysed across all trial sites.
Adaptability to emerging evidence	Adapting to new evidence can be slow, depending on guideline revision cycles and local practice changes.	Rapid adaptation is embedded, with new evidence incorporated into the trial framework for real-time evaluation and adjustment of allocation to various management arms.
Patient and provider burden	Patients and providers bear the responsibility of initiating and sustaining screening discussions.	Burden is reduced by structured invitations and follow-up protocols, minimizing variations in decision-making in screening initiation and follow-up.
Data collection and outcomes tracking	Limited standardized data collection across different providers and sites.	Comprehensive data collection and tracking are embedded in the trial, enabling robust analysis of screening outcomes and pathway effectiveness.
Research and policy integration	Limited research opportunities; local practices may not contribute to broader evidence generation.	Built-in mechanisms for evidence generation support ongoing research, informing local screening policies and future guidelines.

**Table 3:** Example of a possible design of an adaptive platform trial of prostate cancer screening in Canada.^a^

Domain	Description
Population	Men aged 50–69 in participating provinces (with stratification by race, SES, and geography)
Design	Adaptive platform trial embedded in provincial screening infrastructures
Arms at launch	1. Usual Care (control) 2. PSA followed by MRI 3. PSA followed by Microultrasonography 4. PSA + 4Kscore followed by MRI 5. PSA + 4Kscore followed by Microultrasonography 6. PSA + Polygenic Risk Score followed by MRI 7. PSA + Polygenic Risk Score followed by Microultrasonography
Primary outcome	Prostate cancer-specific mortality (long-term); intermediate outcome: metastatic disease at dx
Secondary outcomes	Overdiagnosis, biopsy rates, quality of life, equity indicators
Randomization	Central (leveraging existing population databases), individual-level randomization, stratified by site, age, and risk factors with mailed invitation to participate
Planned adaptations	Add/drop arms based on interim effectiveness, safety, and equity evaluations, as well as emergence of new strategies
Follow-up	Longitudinal, 15+ years; linked to cancer registry and vital statistics

a An adaptive platform trial for prostate cancer screening has already been launched in the UK (TRANFORSM-UK). Multiple elements of this table are inspired by TRANSFORM-UK.

## Conclusion

Canada’s current, opportunistic approach to prostate cancer screening warrants reconsideration. This strategy risks undermining the potential benefits of PSA testing while amplifying harms and deepening disparities, especially among populations that stand to gain the most from early detection. Since the Canadian Task Force's 2014 screening recommendations, advancements in the diagnosis and management of prostate cancer have mitigated the harms of overdiagnosis and overtreatment. A risk-stratified approach is now achievable and could address issues of inequity and low-value screening. Key questions remain about the optimal methods for screening and risk stratification to continue to reduce overdiagnosis and overtreatment. Canada can modernise its approach to prostate cancer screening by implementing programmatic, organised screening through large adaptive platform trials integrated into the healthcare system. This strategy would enable the ongoing integration of emerging evidence and technologies while ensuring equitable access to early detection and standardised management. In doing so, we can ensure that prostate cancer screening effectively serves all Canadians' health.

## Contributors

DDN–Conceptualization, Investigation, Writing–original draft, Writing–review & editing.

AL–Investigation, Writing–review & editing.

CJDW–Investigation, Writing–review & editing.

ARZ–Investigation, Writing–review & editing.

NEF–Investigation, Writing–review & editing.

QDT–Investigation, Writing–review & editing.

AF–Investigation, Writing–review & editing.

LCR–Investigation, Writing–review & editing, Supervision.

ASD–Investigation, Writing–review & editing, Supervision.

MJR–Investigation, Writing–review & editing, Supervision.

GSK–Conceptualization, Investigation, Writing–review & editing, Supervision.

## Declaration of interests

Dr. Girish S. Kulkarni reports serving on advisory boards for Pfizer, AAA/Novartis, Astellas, Verity, Knight Therapeutics, Abbvie, and Bayer, and has received consultancy fees from TerSera and AstraZeneca. All are outside the submitted work.

Dr Lofters reported receiving grant support from Pfizer Inc/ReThink Breast Cancer, outside of the submitted work.

Dr. Christopher J.D. Wallis reports receiving institutional grants from Astellas, Bayer, Knight Therapeutics, and Tolmar Pharmaceuticals. He has received personal consulting fees from Johnson & Johnson Innovative Medicine and Nanostics Inc. He has also received personal honoraria for lectures, presentations, manuscript writing, or educational events from AbbVie, Astellas, AstraZeneca, Bayer, EMD Serono, Haymarket Media, Healing and Cancer Foundation, Johnson & Johnson Innovative Medicine, Knight Therapeutics, Intuitive Surgical, MashUP Media, Merck, Pfizer, Science & Medicine Canada, Sumitomo Pharmaceuticals, TerSera Canada, and Tolmar Pharmaceuticals Canada. Additionally, he has received personal payments for travel or meeting attendance from AbbVie, Tolmar, Knight, and TerSera. All are outside the submitted work.

Dr. Neil E. Fleshner reports receiving academic grants from the Movember Health Equity Grant and the Ontario Institute for Cancer Research (OICR) Pre-Cata Grant. He has received consulting fees from Amgen, Janssen, Astellas, Bayer, Sanofi, Abbvie, and Ferring. He has received honoraria from Astellas for lectures or educational events. He has received support for attending meetings or travel from Nucleix and Point Biopharma. He holds leadership or fiduciary roles with Point Biopharma, Verity Pharmaceuticals, and Soricimed. He also holds stock or stock options in Point Biopharma. All are outside the submitted work.

Dr. Quoc-Dien Trinh reports consulting fees from Astellas, Bayer, Intuitive Surgical, Janssen, Novartis, Pfizer, and research funding from the Pfizer. All are outside of the submitted work.

All other authors report no relevant conflicts of interest.

## Appendix A Supplementary data

French Summary_disclaimer
