# Supplementary material for: Is it time for Canada to revisit its approach to prostate cancer screening?
Source: Lancet Reg Health Am. 2025 Jul 24;49:101180. doi: 10.1016/j.lana.2025.101180 (PMC12485535; doi:10.1016/j.lana.2025.101180)
Supplement: French Summary_disclaimer [file mmc1.docx]

**Editor note :** *This translation in French was submitted by the authors and we reproduce it as supplied. It has not been peer reviewed. Our editorial processes have only been applied to the original abstract in English, which should serve as reference for this manuscript.*

**Résumé**

Le cancer de la prostate est la troisième cause de décès par cancer chez les hommes au Canada. Malgré les progrès réalisés au cours de la dernière décennie pour limiter le surdiagnostic et le surtraitement, les lignes directrices canadiennes recommandent depuis 2014 de ne pas procéder à un dépistage systématique de l'antigène prostatique spécifique (APS). Il en résulte un dépistage opportuniste, marqué par un accès inéquitable, des tests de faible valeur et des occasions manquées de détection précoce. Nous passons en revue les développements mondiaux en matière de politique de la santé, les nouvelles données issues d’essais cliniques et les stratégies de mise en œuvre qui suggèrent qu'un dépistage organisé et stratifié en fonction du risque peut améliorer l’efficacité et l'équité du dépistage du cancer de la prostate. Cependant, le surdiagnostic reste préoccupant dans le cadre des programmes organisés. Pour répondre à cette incertitude et produire en temps utile des données pertinentes pour les politiques, nous proposons de mettre en œuvre des essais plateformes et adaptifs à l'échelle de la population, intégrés au système de santé. Cette approche permettrait l'intégration en temps réel de nouvelles technologies, de protocoles standardisés et d'un accès équitable - caractéristiques essentielles d’un système de santé apprenant. Un tel modèle pourrait aider le Canada à moderniser le dépistage du cancer de la prostate tout en évaluant soigneusement les bénéfices, les risques et l’équité dans un contexte en constante évolution.
